# A Smart Microcontroller Architecture for the Internet of Things

**DOI:** 10.3390/s20071821

**Published:** 2020-03-25

**Authors:** Zhenyu Wu, Kai Qiu, Jianguo Zhang

**Affiliations:** 1 School of Internet of Things, Nanjing University of Posts and Telecommunications, Nanjing 210003, China; 18362997968@163.com; 2 Ordnance N.C.O. Academy, The Army Engineering University of PLA, Wuhan 430075, China; zhjg100@126.com

**Keywords:** smart microcontroller, cloud model, embedded intelligence

## Abstract

The interoperations of endpoint devices are generally achieved by gateways in Internet of Things (IoT) systems. However, the gateways mainly focus on networking communication, which is lack of data logic control capabilities. The microcontrollers with embedded intelligence could work as an intermediate device to help the interconnections of the endpoint devices. Moreover, they could help control the endpoint devices. In this paper, a microcontroller architecture with intelligent and scalable characteristics is proposed. The intelligence means that the microcontroller could control the target endpoint devices by its logical circuits, and the scalability means that the microcontroller architecture could be easily extended to deal with more complex problems. Two real world industrial implementations of the proposed architecture are introduced. The implementations show that the microcontroller is important to provide the intelligent services to users in IoT systems. Furthermore, a simulation experiment based on the cloud model is designed to evaluate the proposed method. The experimental results demonstrate the effectiveness of the proposed architecture.

## 1. Introduction

With the advancements of microelectronics, communications and information technology, the Internet of Things (IoT) concept has been widely applied in various fields, including smart homes [1], health care [2], and things traceability [3]. Nowadays, the academic and industrial studies mainly focus on the following directions, such as IoT system architecture, communication protocols, sensor networks, security and privacy protection and applications of the IoT.

Generally, the IoT refers to the things in physical worlds that could be interoperated with the support of communication protocols [4], and it can be regarded as a network of low-cost electronic devices where sensing data and communication could be achieved automatically. This kind of interoperation among devices is also called “Machine to Machine” (M2M) communication [5], which is important for handling the uncertainty in IoT systems. However, M2M devices are difficult to connect with each other in real world implementations because there is lack of standardized communication protocols. A gateway is typically employed among M2M devices to work as an intermediate device. However, these gateways lack intelligent control capabilities. These capabilities are very important in IoT systems because of the limited computational power and memory of M2M devices. Instead, a microcontroller could directly control the endpoint devices. In addition, it could communicate with the Internet so that intelligent services could be provided to the end users [6].

**Figure 1:**
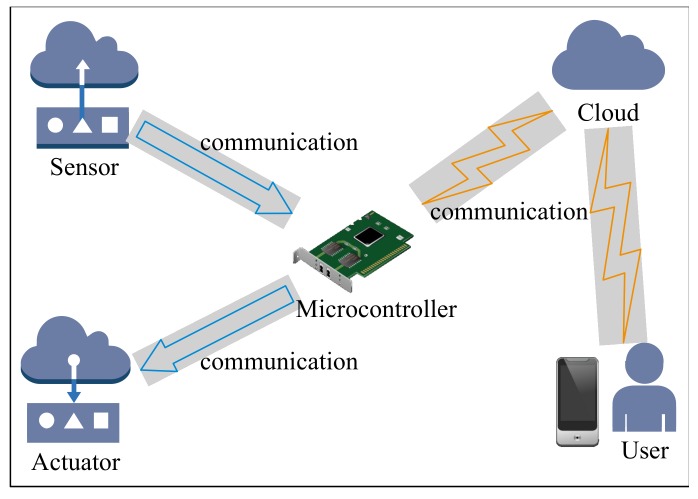
The overview of an IoT system.

An overview of an IoT system is shown in Figure 1, which includes the following key components: (1) sensors: they are used to monitor the environment so that sensing data could be generated [7]. These data could be locally processed or uploaded to the cloud center for secure storage; (2) communication: devices could be interconnected with each other, and have the ability to interconnect with the Internet. Communication protocols such as Zigbee, Bluetooth, and WIFI could be used in this component; (3) microcontrollers: they make all the devices work together as a system to provide the intelligent services. A microcontroller consists of hardware and software components. The sensing data is sent to the microcontroller for processing, and it generates signals for actuators. In this way, the whole system could be controlled to the target state; (4) actuators: these are used to manipulate the environment. Actuators are a kind of transducer device. They takes an electrical signal as an input and convert the signal into a mechanical physical motion; (5) user interfaces: the IoT system should provide intelligent services to its end users, and the interactions between them should be in an appropriate way.

In existing studies, the microelectromechanical systems (MEMS) has been regarded as an valuable technology for the success of the IoT [8] because it could implement a system on a chip with higher performance and lower costs. Therefore, it has been widely applied in sensors [9,10]. Recently, embedded intelligence has been added to sensors for more efficient collaboration [11].

Motivated by this, we argue that the whole IoT system should be incorporated with imbedded intelligence to improve the collaboration efficiency. It is the microcontroller that should be equipped with intelligent techniques to coordinate all components to efficiently work together. In this paper, we focus on the design and implementation of a smart microcontroller for IoT systems. In our opinions, the microcontroller could be regarded as a “brain”, because it can fetch data from sensors, it can analyze data with computational intelligence techniques such as genetic algorithms and neural networks [12], and it can generate the final control commands. Furthermore, the microcontroller architecture should be scalable for different real-world applications. 

In this paper, we illustrate an intelligent and scalable microcontroller architecture for IoT systems. The logical circuit of the microcontroller is responsible for intelligent controls, and a system bus is designed to connect multiple microcontrollers. 

The main contributions are as follows: First, a smart microcontroller architecture is introduced. Furthermore, two industrial IoT systems are implemented to illustrate the applications of the proposed architecture. Both systems include hardware and software implementations. In one system, only one microcontroller is implemented to provide a smart lock service. In another system, multiple microcontrollers are implemented to illustrate the cooperation of microcontrollers for complex tasks. Finally, a simulation experiment is set up. The cloud model which is widely used in artificial intelligence is applied to measure the uncertainties of multiple microcontrollers. Moreover, the system is evaluated using the time cost metric.

The remaining of this paper is organized as follows: the second section describes the Materials and Methods; the third section is the Results; the fourth section is about Discussion of the proposed method. The paper is concluded in the final section.

## 2. Materials and Methods

### 2.1. Related Work

The gateway technology has been widely used in IoT systems. Generally, a gateway is deployed to collect, process, and forward the data received from sensors. In most cases, it plays the same role as a microcontroller. The difference between them is as follows: The gateway mainly focuses on the network connection, which facilitates the seamless integration of IoT systems and the Internet. The microcontroller has much more capacities, such as data mining and logic control.

Naeem et al. designed a wireless gateway which could be regarded as the main component of the IoT architecture [5]. The architecture was composed of three layers: the sensing layers containing the M2M devices; the gateway layer providing the services of network connections; the application layer providing services to users. Mouradian et al. designed a gateway to perform large-scale disaster management applications in IoT systems [13]. In their solution, the gateway is mainly used to connect heterogeneous devices. Moreover, the support of the service portability is also provided. Chen et al. designed an edge gateway, which could be used to achieve networking communication in IoT solutions [14]. Similarly, Abanaie et al. analyzed the performance of IoT gateway [15]. Kelly et al. designed a program in an IoT gateway to transform ZigBee addresses [16]. Moreover, the data payloads of the internet protocol could also be encapsulated by the program. The gateway was used to monitor environmental conditions. Kang et al. investigated IoT application scenarios, and introduced an IoT gateway which could self-configure in real time [8].

Sensors are one of the important components in IoT systems. Currently, intelligent technologies are being incorporated into the design of sensors. This could provide other solutions in IoT systems. Recently, MEMS sensors have been becoming some of the most popular sensors in IoT systems because the can capture data with low energy costs. The applications of the MEMS sensors include smart watches, virtual reality glasses, and fitness trackers [17]. Attaran et al. designed a new RFID tag using MEMS technology. Moreover, MEMS switches are applied to support communication [18]. Wang et al. presented a wireless wearable sensing device based on MEMS for next-generation sports training [19]. The future of the MEMS sensors is to incorporate embedded intelligence [11,20], where the millions of units in MEMS systems could work together to finish a common task. Furthermore, the scalability of the system could be enabled.

IoT technologies with microcontrollers had been applied in various fields, such as wearable systems to remotely monitor the activities of users [21]. In these systems, the main functions of the microcontrollers include data collection, data transfer, and data analysis. Moreover, the microcontrollers are also used to interconnect devices from different vendors. The frequently used microcontrollers include Arduino [22], Raspberry Pi microcontroller board [23], and an 8-bit microcontroller to power lightweight sensors [24]. In industrial applications, the microcontrollers should be well incorporated into the IoT systems. Therefore, the logical circuits would be designed according to the real requirements. Moreover, the distributed architecture might be useful for the complex IoT systems.

The existing studies are mainly focus on the networking aspects, and some studies could achieve basic controls using microcontrollers. However, they have not addressed the intelligence embedded in microcontroller to resolve problems in the industrial applications and have not presented the scalable architectures to solve complex problems. Our work aims at designing a smart microcontroller architecture and illustrating its industrial applications.

### 2.2. The Smart Microcontroller Architecture

With the development of the artificial intelligence technology, a novel smart device is needed to collect, process information in IoT systems, where the microcontroller could be regarded as the “brain”, which could be cooperated with sensors, actuators, and even human beings to achieve much more optimized service provision.

Therefore, the following three principles should be considered when designing a smart microcontroller. First, the microcontroller should be customized. Both the hardware and the software could be customized in different applications. Second, the microcontroller should be smart. It should have the capabilities of data mining and logical control. Third, the microcontroller should be scalable. They could cooperate with each other to accomplish one complex task.

**Figure 2:**
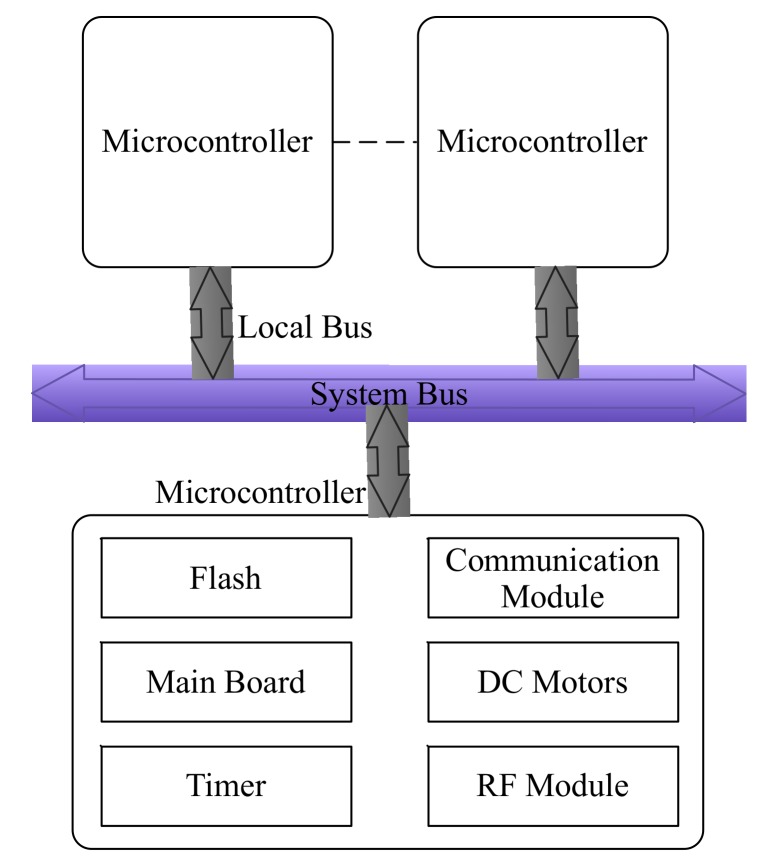
The smart microcontroller architecture.

The proposed smart microcontroller architecture is shown in Figure 2. Generally, a microcontroller includes the following components: main board, flash, timer, communication module, direct current (DC) motors, and radio frequency (RF) modules. The main board is used to connect and coordinate all components. The sensing and configurations data could be stored by the flash component. The timer component could provide the real time of the system. With the communication module, the smart microcontroller could communicate with the remote systems, such as the cloud centers. In order to receive the data from the environment and interact with the environment, two components named DC motors and the radio frequency modules are designed. 

Moreover, a microcontroller should work with other microcontrollers in order to accomplish the complex tasks. This is called the distributed architecture in this paper. In a distributed architecture, the microcontrollers are connected by system bus. Each microcontroller is connected to the system bus by a local bus. The detailed architecture is shown in Figure 2. To handle a complex task in IoT environment, the task should be firstly divided into sub-tasks. Therefore, each sub-task could be controlled by a single microcontroller. All microcontrollers could communicate with each other by means of the system bus mechanism. With the cooperative work of all microcontrollers, the original complex task could be handled.

### 2.3. The Microcontroller Software Framework

The ultimate goal of a smart microcontroller is to provide intelligent services to users. Therefore, in addition to the elaborate design of hardware components, the services that a microcontroller provides should also be clarified. In fact, the ways that a microcontroller provides intelligent services to users is determined by the ways it interacts with other components, which is composed of microcontroller interacting with sensors, microcontroller interacting with actuators, and microcontroller interacting with users.

**Figure 3:**
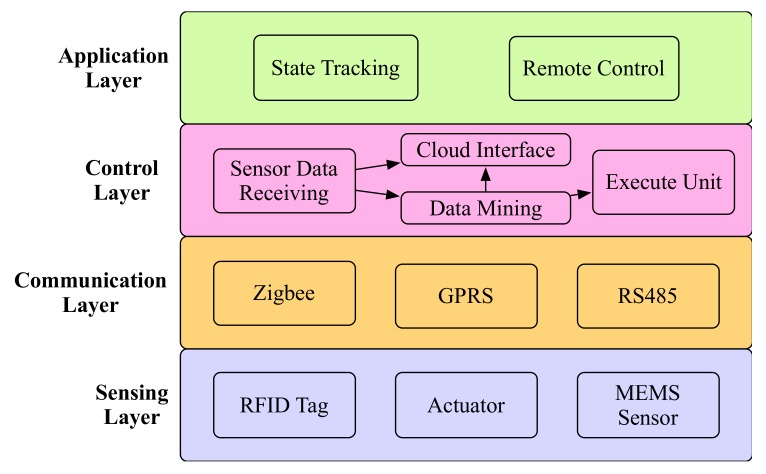
The microcontroller software framework.

The software framework to implement the proposed microcontroller is shown in Figure 3. The functionalities of the microcontroller software are divided into four layers, including the sensing layer, the communication layer, the control layer, and the application layer.

In the sensing layer, the microcontroller interacts with endpoint devices such as sensors and actuators. This layer includes the selection of sensor types according to the different task types, such as RFID tags, MEMS sensors. The sensor data could be regarded as the input of the microcontroller. The actuator receives the output signal of the microcontroller and executes the corresponding actions.

The communication layer includes the communication protocols, which the microcontroller used to interact with other components, such as sensors, actuators, and the cloud centers. Generally, the Zigbee protocol is used to communicate with Zigbee nodes. The GPRS protocol is used to communicate with the remote cloud centers. The RS485 protocol is used to locally communicate with other microcontrollers.

The control layer is the core component of a microcontroller. In this layer, the microcontroller should store and analyze the received data. Therefore, the control layer includes the following functionalities: sensor data receiving, data mining, cloud center interface, and execute unit. The sensor data receiving functionality responds for collecting and storing the sensor data. The data mining functionality is used to analyze the data with machine learning methods, such as the classification and clustering methods. The cloud center interface could send the original data or analyzed results to the remote cloud center. The execute unit fetches the data mining results and determines the output execution commands.

In the application layer, there are two basic functionalities. One is state tracking, and the other is remote control. State tracking could track the status of the sensors, actuators, and the microcontroller itself. The remote control provides a mechanism to remotely control the sensors or actuators.

### 2.4. The Microcontroller Implementations in IoT Systems

The customer requirements should be carefully analyzed before incorporating the proposed architecture into the implementations. First, the hardware interfaces should be designed so that the sensor data could be obtained by the microcontroller. For example, if the communication method of sensors is radio frequency identification (RFID), a RFID reader should be designed and connected to the microcontroller. Second, the logical functions should be implemented in microcontroller. In this way, the sensor data could be processed and the control command would be generated. Finally, the communication method of the microcontroller should be identified. This includes the following two aspects. The one is communication among microcontrollers, and the other is communication between the management software and the microcontroller. The communication protocols, such as RS485 might be used.

## 3. Results

In this section, two industrial implementations of the proposed architecture are introduced. Both of these two implementations are coming from the real requirements of the companies, and they have been successfully deployed for daily uses.

### 3.1. A RFID-Based Microcomputer Lock System Using One Microcontroller

The first implementation has been successfully applied in an electricity company in Jiangsu province of China. In fact, the maintenance of electricity equipment is difficult because of the special characteristics of electricity. Accidents frequently occur when the maintenance workers wrongly operate the equipment. Meanwhile, the amount of electricity equipment has rapidly increased with the development of the power grid, which increases the operation difficulties. Generally, a duplicate naming mechanism could be used to help maintenance workers identify different equipment. However, entering the wrong equipment room or operating the wrong electricity equipment always occurs during the process of their daily maintenance works. In general, a lock system is applied to reliably avoid the occurrence of these accidents. The five anti microcomputer lock is such a solution to effectively avoid accidents involving wrong operations.

However, the five anti microcomputer lock has only 10 bits address space for encoding identification number. That is, it only has 1024 permutations, which will result in the following two drawbacks: first, if the number of pieces of equipment exceeds 1024, the identification numbers assigned to the locks would be duplicated. This would cause the risk of wrong operations. Second, the identification number could only be used at one substation. It could also have the chance to be duplicated at different substations. If the maintenance workers try to sequentially maintain the substations, the digital keys should be repeatedly configured on site to guarantee the uniqueness of the lock identification number. This will waste the time and energy of maintenance workers.

RFID technology is a major breakthrough in the embedded communication paradigm which enables design of microchips for wireless data communication. They help in automatic identification of anything they are attached to acting as an electronic barcode [25]. Therefore, the RFID technology incorporating the proposed microcontroller architecture is applied to effectively solve the above problem. This greatly facilitates the operation and maintenance of the electricity equipment.

**Figure 4:**
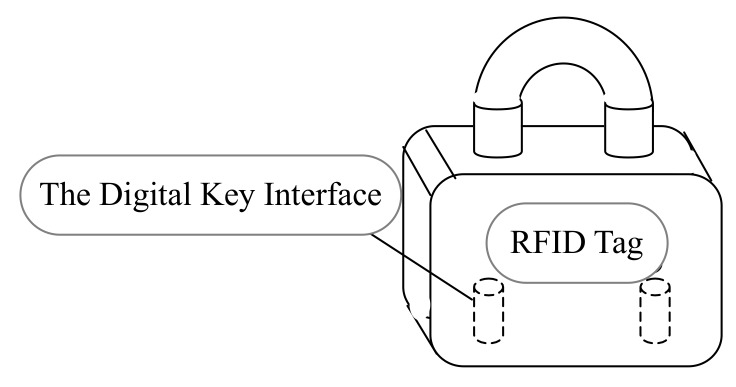
The RFID lock system.

To deploy the product in a more effective way, the original five anti microcomputer lock is improved in the following two steps: first, a RFID tag with a unique identification number is attached to the lock; second, the digital key incorporated the proposed microcontroller architecture is implemented. A RFID reader is implemented in the digital key to decode the identification number from RFID tag. Therefore, the lock could be controlled by RFID tag together with its original machinery. The logical rules are used to determine whether the lock will be unlocked or not. With this solution, the uniqueness of the equipment identification number could be ensured. Generally, 64 bits or 96 bits identification number is enough. Moreover, the remote authentication of the digital key to any equipment could be achieved. The unlock time and the operator of the lock could also be recorded. Therefore the solution could greatly improve the efficiency of maintenance workers and ensure the normal work of the equipment. The system structure is shown in Figure 4. A RFID tag is attached to the lock, and two interfaces are left to the digital key.

To develop a robust system, there are several requirements. First, the system should properly work in the strong electromagnetic environment. Meanwhile, the RFID tag should be safely attached to the surface of the lock. Second, the RFID tag reader could communicate with the RFID tag that is located more than 2 meters away. Third, the digital key should be powered by low power battery. Moreover, it could store data, such as unlock time. Fourth, the digital key could communicate with remote computers by RS232 or USB. Finally, the digital key should automatically shut down in case there are no operations within a specific time, such as 10 min.

**Figure 5:**
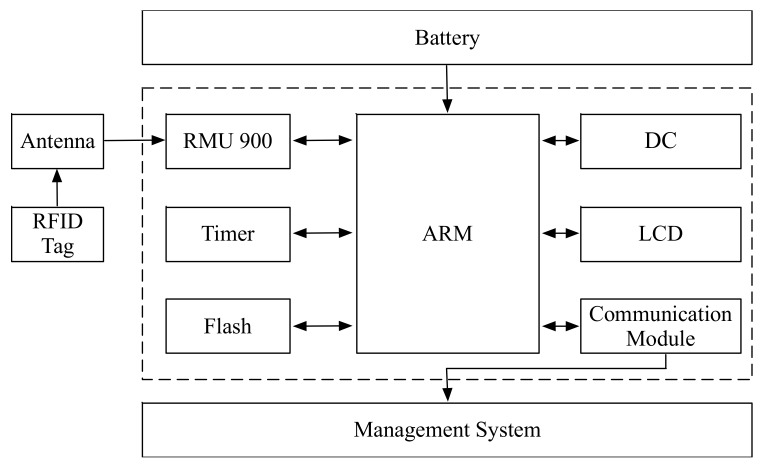
The architecture of digital key in the RFID lock system.

Therefore, the detailed design of the digital key in RFID-based microcomputer lock system is shown in Figure 5. All components have their own functionalities to meet the requirements mentioned above.

#### 3.1.1. RFID Tag Component

A RFID system is composed of tag, reader, and the application software. The tag could also be called transponder. The working flow is as follows: first, the reader emits a radio wave with a specific frequency to the transponder; second, the transponder sends out the data stored in its memory; third, the reader sequentially read the data and send them to the application software. 

There are two types of RFID tags. The one is the passive tag, and the other is the active tag. For the passive tag, after the RFID tag enters the magnetic field, it receives the radio frequency signal sent by the reader. The data stored in the tag chip is sent out with the energy obtained by the induced current. For the active tag, it could send out a signal with a certain frequency. After reading and decoding the data, the reader will send the data to the central information system for further processing.

In order to reduce the maintenance cost, the passive tag with compact size is selected in this system. Moreover, the working frequency of RFID tag is another key point of the whole proposed hardware system because it determines not only the operational principle of the radio frequency identification system, but also the difficulty and cost of the implementation of the RFID reader. In the proposed system, an ultra-high frequency RFID tag with the frequency of 915 MHz is used. Based on this frequency, the RFID tag reader is developed.

#### 3.1.2. RFID Tag Reader Component

In this system, there are two kinds of RFID tag readers. The first one is used to maintain the unique identification number of RFID tag attached to the lock. It could write the encoded identification number to a RFID tag, and it could also correctly read that number. The other one is embedded in the digital key. It could read the encoded identification number from a RFID tag and compare this number with the one stored in the digital lock flash to determine whether the target lock should be unlocked or not.

There are large amounts of adjacent locks in the same equipment room. Therefore, the working distance should be properly set to prevent misreads. 

In our implementation, the RMU900 module is used to work as the key component of the RFID reader. The components such as emission, receiver, radio frequency coupling, and MCU are all integrated in the RMU900. Moreover, it has several advantages. First, an application programming interface (API) is provided to conveniently control the module. Second, it works at low voltage, which is suitable for handheld devices. Based on this module, we implemented the following two functionalities: one is configuring the working frequency and reading distance of the module, the other is reading data from RFID tag and sending it to other circuit for processing and storing.

#### 3.1.3. Central Control Module

The central control module is the core component of the microcontroller. It can read data from flash and perform logical controls. In the digital key, the central control module uses the Acorn RISC Machine (ARM7) processor with advantages of low power consumption and high operation speed.

#### 3.1.4. Battery Component

The digital key is powered by a battery. Therefore, a robust battery is needed to ensure its normal work. Lithium batteries have the advantages of high energy density and long service life (more than 6 years). Moreover, the working voltage of a single battery is 3.7 V or 3.2 V, which shows that it has high power endurance. Furthermore, its self-discharge rate is very low. These advantages show that lithium batteries are one of the best battery solutions for the proposed digital key.

#### 3.1.5. Flash Component

Flash memory is used to store two types of data in the digital key. First, the identification number of the target lock should be stored. Second, other important data such as unlock time, unlock worker should also be stored for future tracking. 

The SST39VF1601 NOR flash memory unit is used in our implemented digital key. It could meet the storage requirements because it can store 2 M of data. The flash address space is shown in Table 1. In this table, not all of the storage contents are listed. Three major contents including working ID, unlock start time, and unlock end time are used to indicate the address space.

#### 3.1.6. Communication Module

The digital key should communicate with the management software so that its status could be tracked. Therefore, the communication module in the digital key has two functionalities. First, setting the configurations of the digital key. Second, showing the running status of the digital key. 

In our implementation, the communication interface is RS232. The format of the communication data is shown in Table 2. The 2-bytes “Start” means the begging of the data frame. The 1-byte “Length” means the length of the whole data frame. The 1-byte “Command” means the control commands sent between the digital key and the management software. The “Data” means real data transferred between the digital key and the management software. The “Checksum” is used to check the correctness of the transferred data.

#### 3.1.7. Management System

The management system includes two software systems. The one is the lock management system, and the other is the remote authentication system.

The lock management system is used to maintain the identification numbers of all the electric equipment. That is, it can assign a unique identification number to electric equipment. Moreover, the unlock time and unlock operator could also be recorded by the management system.

The remote authentication system allows the administrator to remotely authenticate the digital keys. Therefore, one or more locks in different locations could be unlocked. The maintenance workers collect all the equipment needed to be unlocked and send a request to the administrator who will audit the request. If this is a legal request, the identification number will be sent to the digital key. Meanwhile, every operation will be recorded by the system.

**Figure 6:**
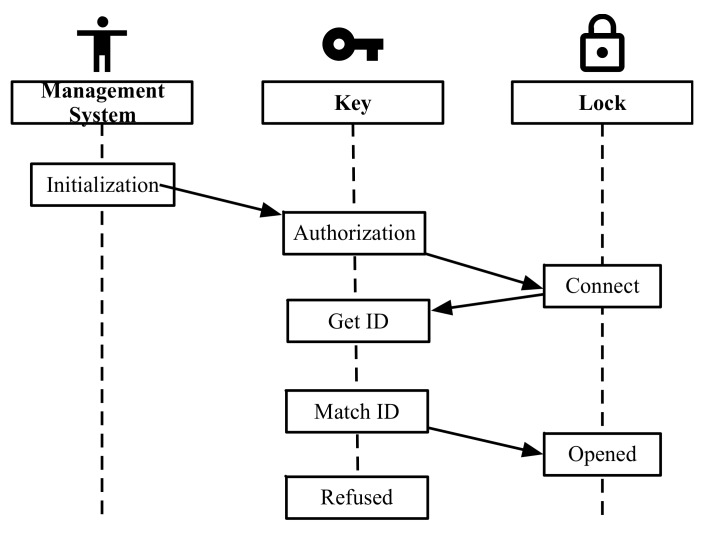
The working flow of RFID lock system.

**Figure 7:**
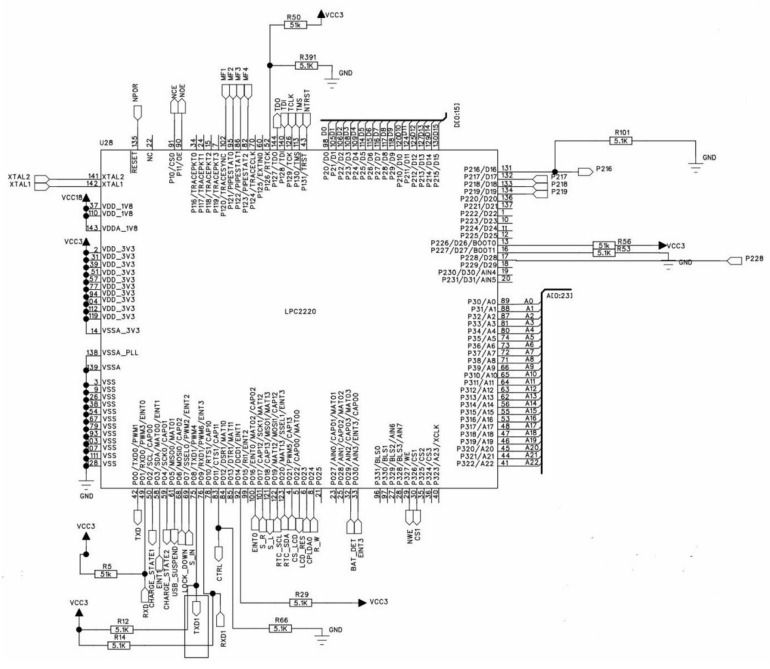
The logical control circuits of the RFID lock system.

The working flow of the RFID-based microcomputer lock system is shown in Figure 6. The first step is “initialization”. A unique identification number generated by management system is assigned to RFID tag which will be attached to the lock. The second step is “authorization”. The maintenance worker submits an unlock request to the administrator according to his working content. The administrator sends the audited identification number to the digital key. The third step is “unlock”. The maintenance worker inserts the digital key into the lock. The digital lock will connect to the lock system and read the identification number. The final step is “match”. If the read identification number is matched with the number stored in the digital key, the lock will be unlocked. Otherwise, the lock cannot be unlocked. The logic control circuits are shown in Figure 7.

**Figure 8:**
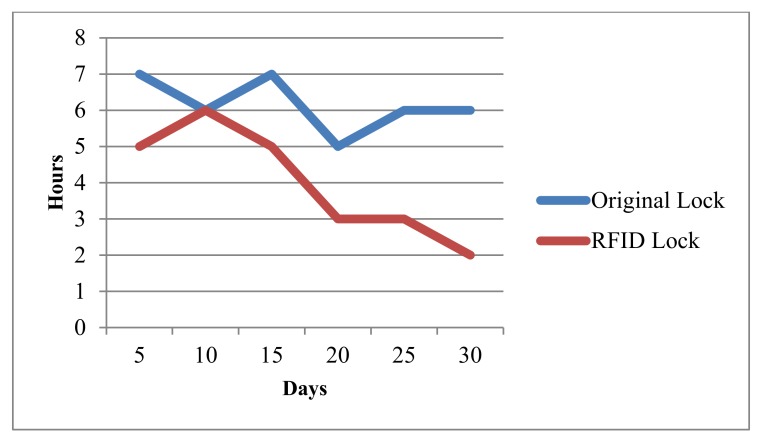
The average maintenance time compared between the RFID lock and the original lock.

The usage data is collected after applying the RFID based microcomputer lock system in the electricity company. The results are shown in Figure 8. In this figure, the horizontal axis stands for the days in one month, and the vertical axis stands for the average maintenance time used in that day. Obviously, the average time is reduced from 7.4 to 4.8 hours when the RFID-based lock is deployed. This result demonstrates that the working efficiency has been improved.

### 3.2. A Distributed Game System Using Multiple Microcontrollers

It is hard to deal with complex problems using a single microcontroller. Therefore, the distributed microcontroller architecture could be used to achieve this goal. In this section, we will show an IoT-based game system with distributed microcontrollers. The real room escape game is originated from a computer game. Players in the room can walk out of the room by answering questions to find the clues.

**Figure 9:**
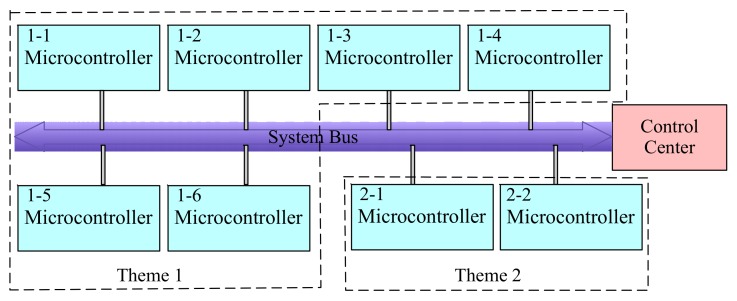
The distributed game system.

There are plenty of themes and scenarios in this game. This requires the system to be scalable. When adding a new scenario, the original system architecture should not be modified. Furthermore, the microcontroller should be reusable. Therefore, the hardware could be reused in each scenario, and the software should be modified for different scenarios. Finally, all microcontrollers should be uniformly controlled for the convenient administration. In order to meet the above requirements, the distributed architecture is designed, as shown in Figure 9. The game has two themes, and every game has different scenarios. Each scenario is assigned to a single room. Therefore, every scenario has a microcontroller called the local controller. All local controllers are connected to a control center by the system bus.

The local controller collects data from different sensors such as light sensor, steering sensor, magnetic induction sensor, RFID tags, and so on. Furthermore, the control signal is generated according to the logical rules embedded in the microcontroller. The control signal will drive other devices including electromagnetic lock, motor, alarm, and audio equipment to finish the control process. In our implementation, the local controller is powered by a 5 V battery. Moreover, it communicates with the control center with a RS485 system bus. With this architecture, the system functionalities could be conveniently extended by connecting a new microcontroller to the RS485 system bus.

The control center is also a microcontroller, which has the same platform with the local controller. Because the control center connects all local controllers, it can remotely control microcontroller of each scenario. For example, the control center could reset every local controller located in the game rooms. Moreover, the control center has management software which could be used by the administrator to remotely control the equipment located in the game room.

**Figure 10:**
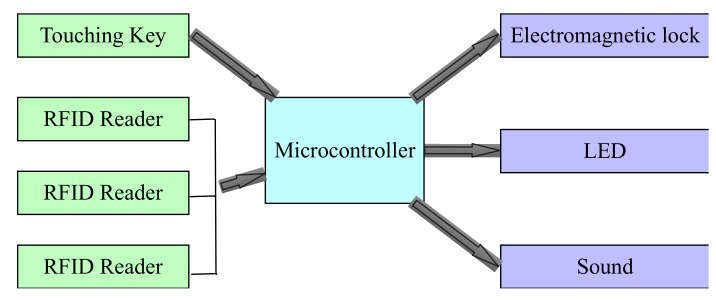
The local controller system.

Because there are lots of local controllers in our implemented distributed game system, but only one local controller is picked up to demonstrate the working flow. The local controller system is shown in Figure 10. In the figure, the left four components are sensors. The touching key is used to capture the touching actions of players. RFID readers are used to receive signals from RFID tags attached in the body of players. The logical computation is executed in the microcontroller, which will generate the control signals. In this example, there are three control signals. One is used to unlock an electromagnetic lock, one is used to control LED light, and one is used to control the sound device.

**Figure 11:**
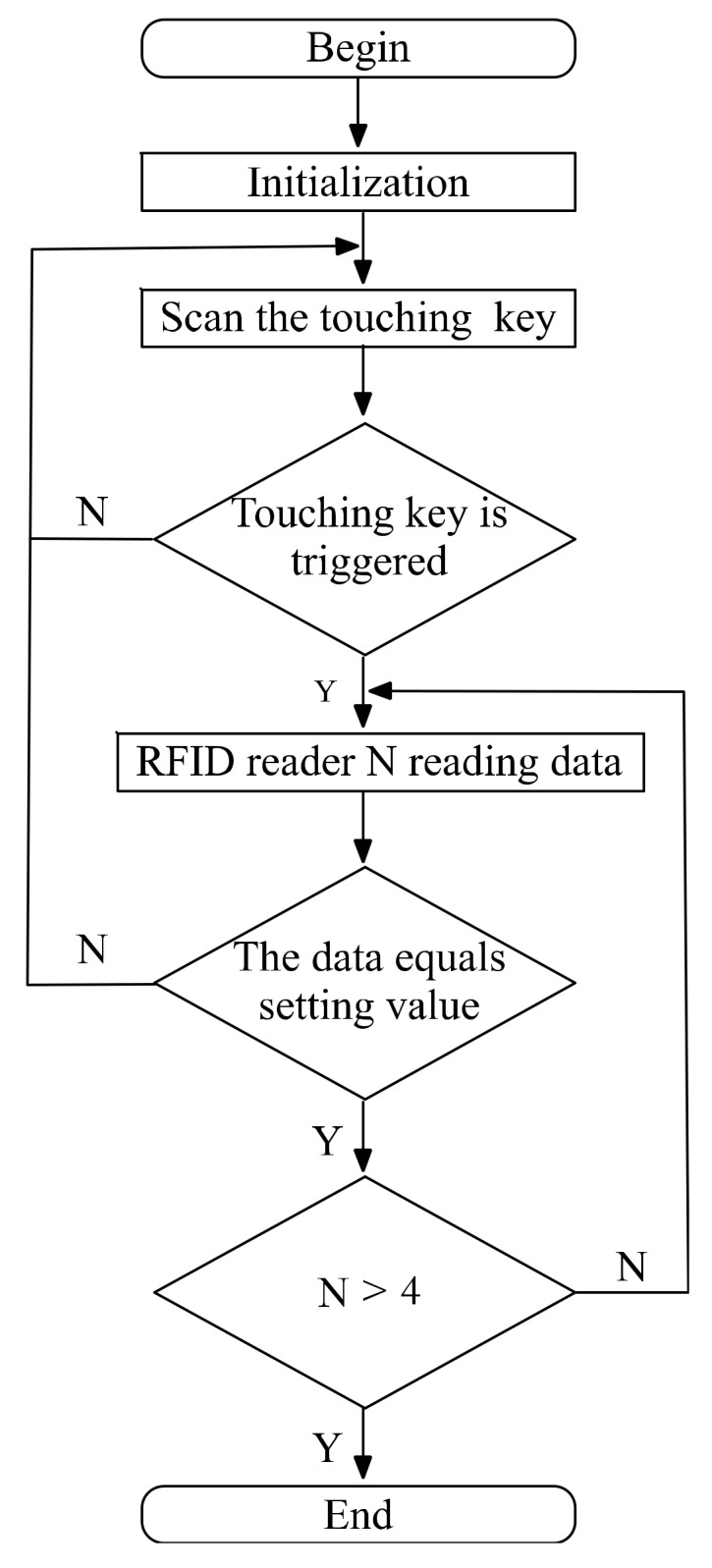
The program flow chart of the local controller.

The program flow chart of the logical control is shown in Figure 11. In initialization step, the configurations of microcontroller are reset. The microcontroller begins to scan the status of the touching key. If the touching key is triggered, it begins to read data from one RFID reader. Otherwise, it will wait for the players to trigger the touching key. The read data is compared with the setting value, and if they are equal, this challenge for the players is passed. When all of the challenges are passed, the program ends.

In summary, a total of eight scenarios are designed in this system. There are 62 buttons, 15 RFID readers and 12 laser sensors are used in the implementation.

### 3.3. Simulation Experiments

To quantitatively evaluate the proposed architecture, a simulation experiment is setup. Assuming that there is a control center C, *M* local centers *LC* = {*S_1_, S_2_, …, S_M_*}. The communication time between the *i*-th local center and C is *t_sci_* (1 ≤ *i* ≤ *M*). The working time between two local centers is *t_ssi_* (1 ≤ *i* ≤ *M* − 1). The processing time of the *i*-th communication request for C is *t_ci_* ~ *f*(M) (1 ≤ *i* ≤ *M*), that is, the time is proportional to *M*. The processing time between the action of a player and the local center is *t_hsi_* (1 ≤ *i* ≤ *M*), and there is *N_i_* actions for the *i*-th local center. The total time *T* used by the system could be defined as follows:

(1) $$T=\overset{M}{\underset{i=1}{\sum }}\left(t_{sci}+N_{i}*t_{hsi}+t_{ci}\right)+\overset{M-1}{\underset{i=1}{\sum }}t_{ssi}$$

where, the former part is used to measure the time costs of communications, and the latter part is used to measure the time costs of a player moving between two local centers. The uncertainties of the simulation experiments are as follows: first, the communication time *t_sci_* is uncertain because different local centers have different communication conditions with the control center. Second, the time *t_ssi_* is uncertain because the time intervals between a player leaving a local center and entering the next local center are different. Finally, the time *t_hsi_* is uncertain because different players have different capabilities to escape from a local center. To simulate the process of the game, the cloud model is applied to handle above uncertainties. The cloud model is a model of the uncertainty transition between a linguistic term of a qualitative concept and its numerical representation, which could deal with fuzziness and uncertainties in a unified way. It can be explained by means of the traditional probability and statistics theory and fuzzy set theory [12]. The cloud model has been widely used in data mining, nonlinear systems control, knowledge discovery, subjective trust management systems and evolutionary computing etc.

**Figure 12:**
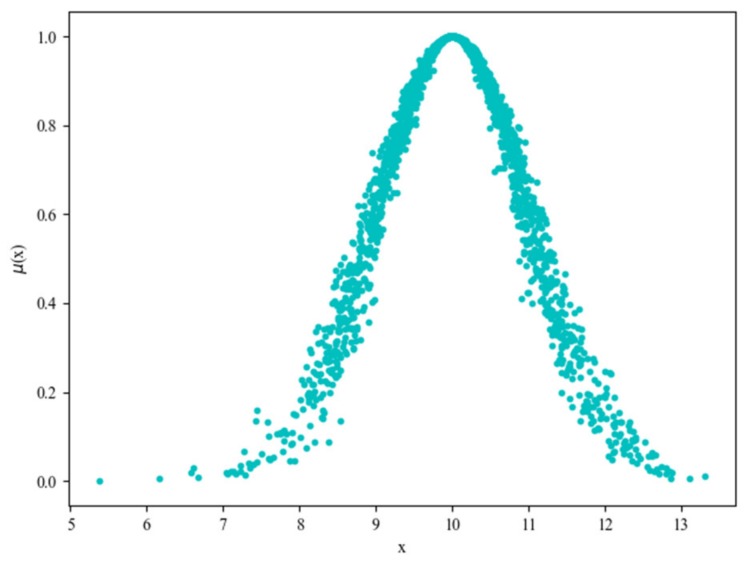
The normal cloud model.

Normal distribution is one of the most important distributions in probability theory. It is usually represented by two numeric characteristics: mean and variance. The normal cloud model is proposed based on the normal distribution. It has three numeric characteristics: Expected value (*Ex*), Entropy (*En*) and Hyper-Entropy (*He*). *Ex* is the position corresponding to the center of the cloud gravity, whose elements are fully compatible with the linguistic concept, *En* is a measure of the concept coverage, *He* is a measure of the dispersion on the data samples, which can also be considered as the entropy of *En*. Vector *v* = (*Ex, En, He*) is called the eigenvector of a cloud. With the given algorithms of forward and backward cloud generators, it is easy to build the mapping between qualitative concept and quantitative data. The forward cloud generator algorithm generates the data samples by inputting (*Ex, En, He*). On the contrary, the backward cloud generator algorithm calculates three numeric characteristics (*Ex, En, He*) by processing data samples. A normal cloud model is shown in Figure 12, the data is generated using a forward cloud generator with three input parameter *Ex* = 10, *En* = 1, *He* = 0.1.

The normal cloud model is used to handle the uncertainties in simulations by using the normal cloud (0.9, 0.2, 0.02) to measure *t_sc_*, using the normal cloud (0.3, 0.05, 0.01) to measure *t_hs_*, using the normal cloud (3, 1, 0.1) to measure *t_ss_* and using the normal cloud (0.5, 0.05, 0.01) to measure *t_c_*. The parameters are listed in Table 3.

**Figure 13:**
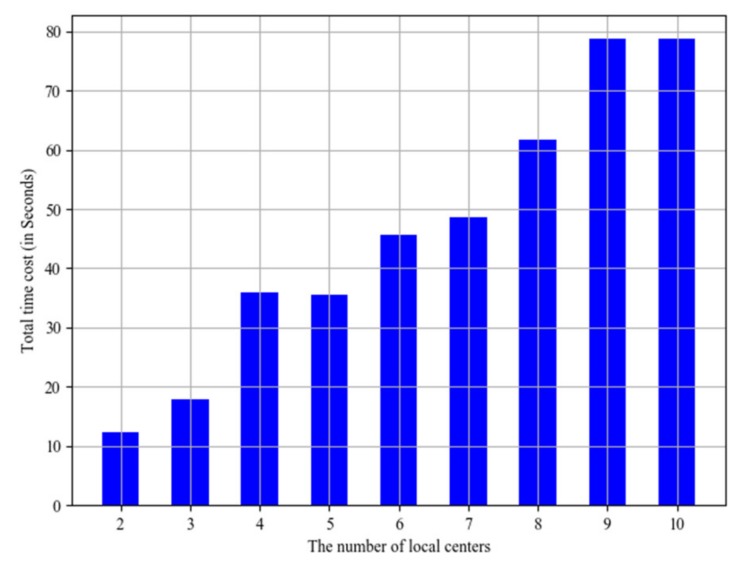
Total time costs with different local center number.

The number of local centers is increasing to evaluate the scalability of the system. The total time costs with different local center number are shown in Figure 13, where the horizontal axis stands for the number of local centers, and the vertical axis stands for the corresponding total time cost. Obviously, the total time costs are increasing with the growth of the local center number. This shows that the player has spent much more time because of the increase of local center number. Moreover, the system is extendable.

**Figure 14:**
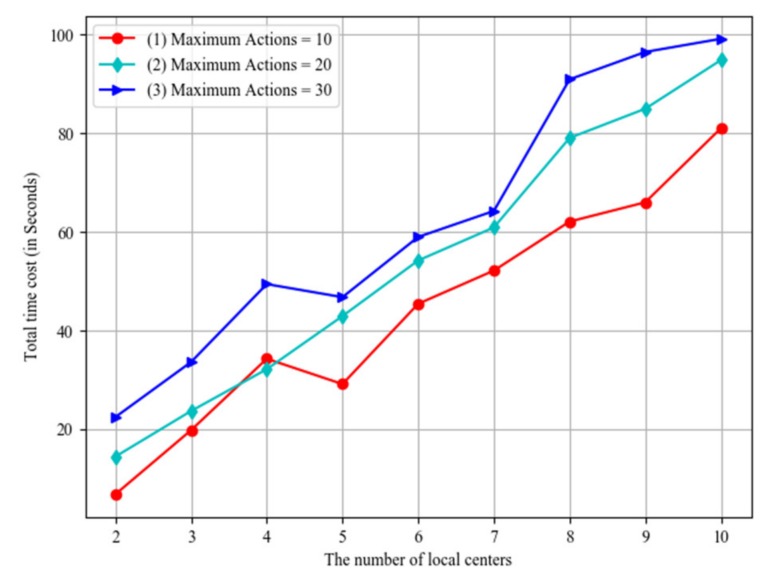
The impact of player actions.

During the process of the game, a player would have interactions with the control system. The action number shows the capability of the player. That is, the level of the player is not high if he has used too many actions. The impact of the player actions is shown in Figure 14, where the horizontal axis stands for the number of local centers, and the vertical axis stands for the total time costs. Line (1), line (2) and line (3) respectively show the results with maximum actions equaling 10, 20 and 30. The results demonstrate that the higher level of the player, the less time he would use.

**Figure 15:**
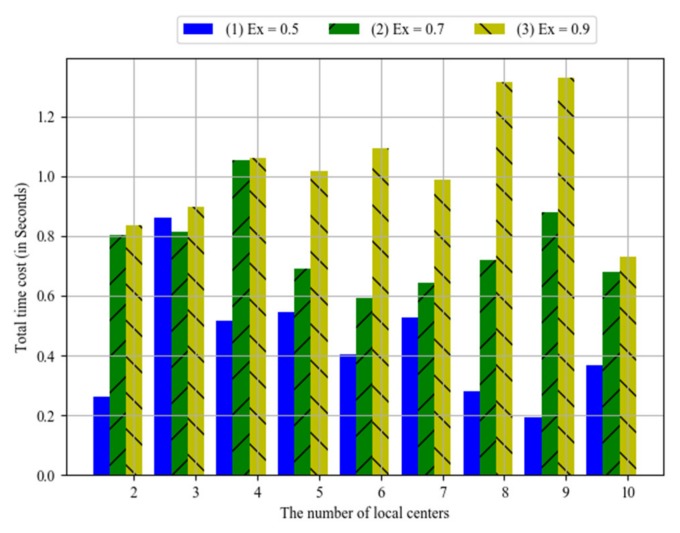
The impact of system bus communication.

The system bus is very important for the communication between local centers and the control center. Its communication time might be affected by network conditions. Parameter *Ex* could reflect different network conditions. For example, it would be a higher value for more congested network. The simulation results are shown in Figure 15, where the horizontal axis stands for the number of local centers, and the vertical axis stands for the total time costs. Bar (1), bar (2) and bar (3) respectively show the results when *Ex* equals 0.5, 0.7 and 0.9. Obviously, the total time costs increase with the values of *Ex*. That is, the communication time of system bus is greater, the total time costs would be greater.

**Figure 16:**
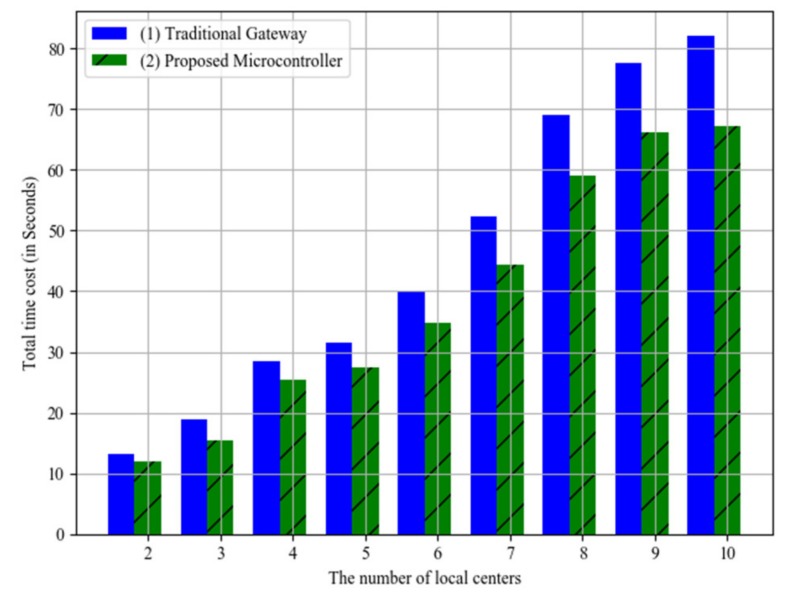
Efficiency comparations with traditional gateway.

The efficiency comparations between the proposed microcontroller and the traditional gateway are shown in Figure 16, where the horizontal axis stands for the number of local centers, and the vertical axis stands for the total time costs. In this simulation experiments, we assume that the traditional gateway only has network functions instead of intelligent data processing functions. The time costs of the proposed microcontroller are lower than those of the traditional gateway. The maximum difference value is 14.89 seconds when the local center number is 10, and the maximum difference value is 1.32 seconds when the local center number is 2. Moreover, the average difference value is 6.84 seconds. The average time saving rate is about 17.5%. This is because of the parallel working of the microcontrollers in the proposed architecture. Instead, the traditional gateway would send the data to control center for further processing.

## 4. Discussion

The advantages of the proposed microcontroller architecture are as follows: First, the microcontroller is intelligent. It could work with the endpoint device and make intelligent control. Second, the microcontroller is scalable. As the IoT systems are uncertain, the whole system should be easily extended when a new requirement arrives. Third, the microcontroller is cost-effective. The implementation could use the existing platform, such as ARM. Moreover, because each microcontroller could concurrently finish a separate task, the proposed architecture has much more efficiency. Therefore, it could be applied in industrial implementations. There are also some disadvantages in the proposed architecture. In our current implementations, communication methods, such as RS483, are used. The communication approach should be extended to other wireless approaches so that much more flexible applications could be developed in IoT systems.

## 5. Conclusions

IoT technologies have changed our daily life. More and more intelligent services will be provided to users by IoT systems. In this paper, we think that the IoT should be regarded as a system in which not only the sensor technologies but also the microcontroller technologies are important components. Moreover, as the artificial intelligence technologies are widely used, all components have the chance to be intelligently designed to cooperate much more efficiently so that the better services could be provided to users.

In this background, this paper studied the smart microcontroller technologies for IoT systems. The main contributions are as following two aspects. First, a microcontroller should be intelligent. It should work as the “brain” in the IoT system. Second, a microcontroller architecture should be scalable. A new microcontroller should be easily extended to the original system for the complex tasks. 

Two industrial implementations are introduced to illustrate the proposed architecture. Both implementations have been deployed in companies for daily uses and obtained better performance. Moreover, the second implementation is simulated using a cloud model which is very popular in the domain of artificial intelligence with uncertainty. The simulation results demonstrate the effectiveness of the proposed architecture.

## Figures and Tables

**Table 1:** The address space of flash.

Address	Content	Length (Bytes)
0	Working ID	4
4	Unlock Start Time	7
11	Unlock End Time	7

**Table 2:** The communication data frame format.

Start	Length	Command	Data	Checksum
2 bytes	1 byte	1 byte	0-n byte	1 byte

**Table 3:** Parameters of the cloud model.

Time Items	*Ex*	*En*	*He*
*t_sc_*	0.9	0.2	0.02
*t_hs_*	0.3	0.05	0.01
*t_ss_*	3	1	0.1
*t_c_*	0.5	0.05	0.01
